# Supplementary material for: Modeling glioblastoma heterogeneity as a dynamic network of cell states
Source: Mol Syst Biol. 2021 Sep 16;17(9):e10105. doi: 10.15252/msb.202010105 (PMC8444284; doi:10.15252/msb.202010105)
Supplement: Supplementary file 5 — Source Data for Figure 3 [file MSB-17-e10105-s001.zip › Figure3A_sourcedata/GSEA_3065/nosigdb_state5.GseaPreranked.1622718435914/SUVA_OPC_PMID_31327527.html]

Details for gene set SUVA\_OPC\_PMID\_31327527[GSEA]

|  || Dataset | state5 |
| Phenotype | NoPhenotypeAvailable |
| Upregulated in class | na\_pos |
| GeneSet | SUVA\_OPC\_PMID\_31327527 |
| Enrichment Score (ES) | 0.58054644 |
| Normalized Enrichment Score (NES) | 1.968362 |
| Nominal p-value | 0.0 |
| FDR q-value | 0.0018231143 |
| FWER p-Value | 0.011 |
Table: GSEA Results Summary

  

Fig 1: Enrichment plot: SUVA\_OPC\_PMID\_31327527      
 Profile of the Running ES Score & Positions of GeneSet Members on the Rank Ordered List

  

| PROBE | GENE SYMBOL | GENE\_TITLE | RANK IN GENE LIST | RANK METRIC SCORE | RUNNING ES | CORE ENRICHMENT || 1 | PTPRZ1 |  |  | 12 | 0.862 | 0.0709 | Yes |
| 2 | BCAN |  |  | 15 | 0.850 | 0.1420 | Yes |
| 3 | SCRG1 |  |  | 36 | 0.715 | 0.1996 | Yes |
| 4 | GPM6B |  |  | 41 | 0.702 | 0.2580 | Yes |
| 5 | PGRMC1 |  |  | 52 | 0.639 | 0.3105 | Yes |
| 6 | TTYH1 |  |  | 141 | 0.489 | 0.3417 | Yes |
| 7 | SCD5 |  |  | 173 | 0.460 | 0.3768 | Yes |
| 8 | VCAN |  |  | 191 | 0.444 | 0.4121 | Yes |
| 9 | ALCAM |  |  | 225 | 0.422 | 0.4438 | Yes |
| 10 | PCDHGC3 |  |  | 428 | 0.331 | 0.4490 | Yes |
| 11 | P2RX7 |  |  | 473 | 0.319 | 0.4708 | Yes |
| 12 | SERINC5 |  |  | 477 | 0.318 | 0.4971 | Yes |
| 13 | THY1 |  |  | 546 | 0.299 | 0.5146 | Yes |
| 14 | CADM2 |  |  | 739 | 0.253 | 0.5143 | Yes |
| 15 | RNF13 |  |  | 745 | 0.251 | 0.5348 | Yes |
| 16 | OLIG1 |  |  | 809 | 0.239 | 0.5478 | Yes |
| 17 | LMF1 |  |  | 832 | 0.235 | 0.5650 | Yes |
| 18 | TMEM206 |  |  | 886 | 0.228 | 0.5782 | Yes |
| 19 | PLP1 |  |  | 1022 | 0.208 | 0.5805 | Yes |
| 20 | NLGN3 |  |  | 1947 | 0.115 | 0.4870 | No |
| 21 | FIBIN |  |  | 2599 | 0.074 | 0.4204 | No |
| 22 | PHYHIPL |  |  | 2821 | 0.062 | 0.4008 | No |
| 23 | FXYD6 |  |  | 2847 | 0.060 | 0.4031 | No |
| 24 | EPB41L2 |  |  | 2855 | 0.060 | 0.4073 | No |
| 25 | CA10 |  |  | 3556 | 0.025 | 0.3312 | No |
| 26 | SIRT2 |  |  | 3589 | 0.024 | 0.3296 | No |
| 27 | CNTN1 |  |  | 4200 | -0.001 | 0.2615 | No |
| 28 | TNS3 |  |  | 4375 | -0.009 | 0.2429 | No |
| 29 | RTKN |  |  | 5585 | -0.057 | 0.1125 | No |
| 30 | FGF12 |  |  | 6306 | -0.091 | 0.0396 | No |
| 31 | LIMA1 |  |  | 6339 | -0.092 | 0.0438 | No |
| 32 | PSAT1 |  |  | 7709 | -0.220 | -0.0908 | No |
| 33 | PMP2 |  |  | 8058 | -0.288 | -0.1055 | No |
| 34 | CNP |  |  | 8294 | -0.352 | -0.1023 | No |
| 35 | FABP5 |  |  | 8829 | -0.684 | -0.1046 | No |
| 36 | RAB31 |  |  | 8831 | -0.687 | -0.0471 | No |
| 37 | DBI |  |  | 8895 | -0.764 | 0.0099 | No |
Table: GSEA details [plain text format]

  

Fig 2: SUVA\_OPC\_PMID\_31327527: Random ES distribution      
 Gene set null distribution of ES for **SUVA\_OPC\_PMID\_31327527**
